# Natural and Modified Montmorillonite Clays as Catalysts for Synthesis of Biolubricants

**DOI:** 10.3390/ma11091764

**Published:** 2018-09-18

**Authors:** Francisco Murilo Tavares Luna, Juan Antonio Cecilia, Rosana Maria Alves Saboya, Deicy Barrera, Karim Sapag, Enrique Rodríguez-Castellón, Célio Loureiro Cavalcante

**Affiliations:** 1 Grupo de Pesquisa em Separações por Adsorção, Departamento de Engenharia Química, Universidade Federal do Ceará, Campus do Pici, Bl. 709, 60.455-900 Fortaleza CE, Brazil; murilo@gpsa.ufc.br (F.M.T.L.); rosana@gpsa.ufc.br (R.M.A.S.); 2 Departamento de Química Inorgánica, Cristalografia y Mineralogía, Facultad de Ciencias, Universidad de Málaga, Campus Teatino, 29071 Málaga, Spain; jacecilia@uma.es (J.A.C.); castellon@uma.es (E.R.-C.); 3 Laboratorio de Sólidos Porosos, INFAP, CONICET, Universidad Nacional de San Luis, 5700 San Luis, Argentina; deicybarrera@gmail.com (D.B.); sapag@unsl.edu.ar (K.S.)

**Keywords:** bentonite, montmorillonite, pillared clays, esterification, castor oil, biolubricants

## Abstract

In this study, natural and modified clays were evaluated as catalysts in an esterification reaction to obtain bio-based lubricants. The biolubricants are environmentally preferred to petroleum-based lubricants because they are biodegradable and non-toxic. Other advantages include very low volatility due to the high molecular weight and excellent viscosity properties with temperature variations. Modifications in natural clay were performed intending to obtain materials with different textural properties that could improve the reaction under study. The modified clays were obtained in two ways: by pillarization using Al_13_ Keggin polyoxocations or by acid treatments with H_2_SO_4_, HCl and HNO_3_. All samples were evaluated for the esterification reaction of fatty acids from castor oil (FACO) using 2-ethyl-hexanol. During the reaction step, a zeolite-based adsorbent was used for water removal to increase the reaction equilibrium conversion. Gas chromatography and nuclear magnetic resonance were performed to ensure the formation of the products. The highest conversion of fatty acids to esters was obtained using pillared clays. Adding adsorbent in the reaction medium (10 g of 3A zeolite to 100 g of FACO), the conversion improved from 74–88 wt % after 6 h at 50 °C.

## 1. Introduction

The depletion of fossil fuels has led to the research and development of more environmentally-benign alternative energy sources since petroleum-based lubricants may pollute the air due to their volatility, as well as contaminate the soil, which can seriously affect health [1,2]. Several studies have reported that the inhalation of petroleum-based lubricants may be potentially carcinogenic [3].

Biomass residues are unique raw materials that can replace fossil fuels to synthesize both fuels and chemicals. Biolubricants are compounds that are more readily biodegradable and non-toxic, so these products lack danger to the environment [2,4,5]. In addition, biolubricants also display several performance benefits such as a higher viscosity index, lower volatility, higher flash point, better lubricity, higher char stability, higher resistance to humidity, lower compressibility or higher dispersancy [2,4,6,7,8,9] in comparison to traditional petroleum-based lubricants. However, these biolubricants also display several drawbacks, such as their poor thermal performance and low thermal oxidative stability [2,5], but these properties may be improved by chemical modifications [5] to obtain biolubricants with applications in several fields [10].

Biolubricants may be obtained from several plants such as sunflower, soybean, palm, rapeseed, coconut or castor [11]. Most of the possible raw materials for the synthesis of biolubricants compete with the food chain, so it is necessary the search for plants that are not intertwined with the food chain. Castor oil is a non-edible oil with interesting physicochemical properties due to its high content of ricinoleic acid (~90 wt %). This compound contains a -OH group in the 12 position providing a higher viscosity and boiling point in comparison to other free fatty acids [5,12]. Nonetheless, ricinoleic acid also displays disadvantages related to its faster oxidation of the unsaturations due to its poor thermo-oxidative stability [5,13].

The esterification reaction of ricinoleic acid with branched alcohols is a process frequently used to improve the physicochemical properties of biolubricants. Traditionally, the main methodologies for the synthesis of esters described in the literature are associated with the reaction between vegetable oils, or fatty acids, with short chain alcohols through homogenous catalysis in the presence of strong inorganic acids, such as H_2_SO_4_ or HCl [14,15,16,17]. Despite the higher conversion values, these mineral strong acids are harmful to the environment, requiring more costly processes in product separation/reaction medium and neutralization steps to be discarded in wastewater; besides that they may cause corrosion to process equipment [18]. Heterogeneous solid acid catalysts have emerged as an alternative since they display several advantages such as easy regeneration, non-toxic characteristics, as well as faster and more efficient separation steps [19]. The need to find economically-competitive solid acid catalysts to obtain large proportions of esters at relatively low cost has led to the development of inexpensive active phases. In this sense, clay mineral-based catalysts are noteworthy raw materials due to their abundance, versatility, the potential to modify their textural properties and their environmental inertness [20].

The modification of textural and chemical properties by acid treatments is the easiest and most inexpensive strategy to increase the specific surface area and pore volume of the raw clay minerals, as well as to remove impurities in clay, favoring their use as heterogeneous catalysts due to the formation of acid sites and higher thermal stability [21]. The acid treatment consists of the activation of the clay mineral by washing of the material under acid conditions, causing the formation of cavities by a partial leaching of the Mg^2+^ and Al^3+^ ions located on the octahedral positions of the clay mineral [22]. Furthermore, this process favors the relocation of Al^3+^ species in the interlayer spacing, leading to this species acting as acid sites [23]. The acid treatment promotes an increase in Brönsted sites’ concentration, since the leaching of the Al^3+^ species provokes a decrease of the number of Lewis sites, while the silanol groups, attributed to the Brönsted sites, are maintained [24]. The use of acid treatment allows the modification of the amount of acid centers in the clay-based materials. Nonetheless, the temperature of the treatment and the acid concentration must be controlled since the use of severe conditions may collapse the clay framework [20,22]. It has been reported in the literature that the use of low concentrations of strong acids as H_2_SO_4_, HCl or HNO_3_ confers Brönsted sites to the clay mineral [22,24].

The textural properties and the amount of acid sites of montmorillonite may be improved by the insertion of pillars between adjacent layers. The insertion of polyoxocations, as the Keggin ion, in the interlayer space increases the interlayer spacing of the smectites from 9.6–18.9 Å [20]. Upon intercalation, the materials are calcined for the metal oxide pillars’ formation between the clay layers by the dehydration and dehydroxylation of the polyoxocations. The formation of these pillars increases the thermal stability of the obtained material with respect to the natural clay and also generates microporosity in the final material [20].

Also recognized in the literature, a molecule of fatty acid reacts with an alcohol molecule to form an ester molecule and water as a by-product in the esterification reaction. The water removal obtained during the reaction favors the reaction equilibrium displacement towards the formation of products, improving the conversion values [25]. The water removal under the esterification reaction conditions, i.e., atmospheric pressure and the temperature relatively lower (T < 100 °C), is only possible using a water-selective adsorbent. In this sense, 3A zeolite is an appropriate material to adsorb H_2_O of the reaction medium due to its average pore diameter, which facilitates the water molecules’ penetration and adsorption in its framework [26].

In this study, natural and modified clays were evaluated as catalysts in the esterification reaction of the fatty acids from castor oil (FACO) with 2-ethylhexanol. Esters with long chains are biodegradable and non-toxic in comparison to petroleum-based lubricants. The high molecular weight provides low volatility, and excellent viscosity properties with temperature variations are other factors to be considered. The esterification reactions using natural and modified clays as catalysts were studied by varying the molar ratio fatty acid/alcohol and the relative amount of catalyst and adsorbent with respect to the fatty acid, trying to maximize the equilibrium conversion of the esterification reaction.

## 2. Materials and Methods 

### 2.1. Materials

Bentonite from Alto Valle (Neuquen, Argentina) was used as the starting material. This clay mineral has shown a cation exchange capacity of 0.89 meq·g^−1^ [27]. The AlCl_3·_6H_2_O and NaOH produced by J.T. Baker (Phillipsburg, NJ, USA) were used to prepare the pillaring solution. The natural and pillared interlayered clays (PILC) were treated with sulfuric acid (H_2_SO_4_, 98 wt %), hydrochloric acid (HCl, 37 wt %) and nitric acid (HNO_3_, 65 wt %) (Vetec, Duque de Caxias, Brazil). Samples of fatty acid from castor oil (FACO) were supplied by Miracema-Nuodex (Campinas, Brazil). This acid contains the following composition: ricinoleic acid (C18:1-OH12) 85.4 wt %, linoleic acid (C18:2) 6.6 wt %, oleic acid (C18:1) 5.3 wt %, palmitic acid (C16:0) 1.5 wt % and stearic acid (C18:0) 1.2 wt %. The calculated mean molar mass of FACO was 295.62 g mol^−1^. 2-ethylhexanol (EH) was supplied by Sigma-Aldrich (Saint Louis, MO, USA). Commercial 3A zeolite was obtained from Grace (Columbia, MD, USA) in a spherical shape. The particle sizes, obtained by the Tyler/mesh procedure, were between 1.68 and 2.38 mm. The gases employed were He (Air Liquide 99.99%), N_2_ (Air Liquide 99.9999%), Ar (Air Liquide 99.99%) and NH_3_ (Air Liquide 99.9%). 

### 2.2. Preparation of Pillared Interlayered Clays (PILCs)

The first step of the pillaring process is to prepare the pillaring agent. The pillaring agent was an aluminum polyoxocation prepared by the basic hydrolysis of an aluminum salt. A 0.5 M NaOH solution was slowly added over a 0.2 M AlCl_3_ 6H_2_O solution in sufficient quantity to obtain a molar ratio OH^−^/Al^3+^ = 2. This mixture was stirred at 60 °C for 1 h and then kept under stirring for 12 h at room temperature (25 °C). The solution used for pillaring contains Al_13_ Keggin polyoxocations (precursors of the pillars) that can be ion exchanged with the charge-compensating cations of the montmorillonite clay. The pillaring solution was added drop-wise to a montmorillonite suspension of 3 g of the clay per 100 mL of water under magnetic stirring for 1 h and later aged for 12 h at the same temperature. Afterwards, the samples were washed several times with distilled water using a dialysis membrane (INLAB Confiança, São Paulo, Brazil), dried at 60 °C overnight and calcined at 500 °C for 1 h to obtain the pillared materials.

### 2.3. Acid Treatment

The bentonite sample was modified with acid treatment using sulfuric acid (H_2_SO_4_), nitric acid (HNO_3_) and hydrochloric acid (HCl). Five grams of bentonite were dispersed in 100 mL of 2.5 M acid solutions and stirred at room temperature for 48 h. Then, the bentonite was separated by centrifugation and washed to remove the mineral acid until a neutral pH was reached. Finally, the bentonites treated with acid solutions were dried at 80 °C for 2 h.

The samples were labeled as follows: NB, for natural bentonite; B-H_2_SO_4_, for bentonite treated with H_2_SO_4_; B-HCl, for bentonite treated with HCl; and B-HNO_3_, for bentonite treated with HNO_3_.

### 2.4. Catalyst Characterization

X-ray powder patterns were collected using a Philips X’Pert Pro MPD automated diffractometer (PANalytical B.V., Amsterdam, The Netherlands) equipped with a Ge(111) primary monochromator (strictly monochromatic CuKα1 radiation) and an X’Celerator detector, working at 45 kV and 40 mA. The powder patterns were recorded between 10 and 70° in 2θ with a step of 0.017°.

The chemical compositions of all clay samples were carried out using the MagiX X-ray fluorescence (XRF) spectrometer of PANalytical.

N_2_ adsorption-desorption isotherms at −196 °C were carried out using volumetric adsorption equipment (AUTOSORB-1MP, Quantachrome Instruments, Boynton Beach, FL, USA). Samples were previously degassed at 150 °C for 12 h. The specific surface area of the samples was estimated using the Brunauer, Emmet and Teller (BET) method [28], and the total pore volume was obtained from the N_2_ adsorption data at relative pressure of 0.95. Micropore volume (V_µP_) was evaluated with the α_S_-plot method using the natural clay and pillared clay reference materials reported elsewhere [29].

FT-IR spectra were registered on a Varian 3100 FT-IR (Palo Alto, CA, USA) spectrophotometer. The interferograms consisted of 200 scans, and the spectra were collected using a KBr spectrum as the background. For each spectrum, about 30 mg of finely ground catalyst were placed in the sample holder.

The amount of acid sites was evaluated from ammonia thermoprogrammed desorption in a home-made system (NH_3_-TPD) profiles. In a typical test, 0.080 g of sample were placed into a tubular reactor; later, the sample was cleaned in He flow (35 mL·min^−1^) from room temperature to 550 °C (maintained for 10 min and cooling to 100 °C). Ammonia adsorption was carried out at 100 °C for 5 min. Helium was then passed at 35 mL·min^−1^ to eliminate physisorbed ammonia. Finally, thermoprogrammed desorption was carried out by heating the samples from 100–550 °C at a heating rate of 10 °C min^−1^. The evolved ammonia was analyzed by an on-line TCD.

FTIR spectra of adsorbed pyridine were recorded on a Shimadzu 8300 FTIR (Kyoto, Japan) spectrometer at a resolution of 4 cm^−1^. Each spectrum was averaged over 128 scans. Self-supporting wafers of the samples with a weight/surface ratio of about 12 mg·cm^−2^ were placed in a vacuum cell with greaseless stopcocks and CaF_2_ windows. The samples were evacuated at 350 °C and 10^−4^ Torr overnight, exposed to pyridine vapors for 15 min at room temperature and then degassed at different temperatures. The net FTIR spectra of adsorbed pyridine were obtained after subtracting the background spectrum of the solid.

The quantification of the Si^4+^, Al^3+^ and Mg^2+^ species, leached during the catalytic test, was determined by ICP-MS using a Perkin Elmer spectrophotometer (NexION 300D, Waltham, MA, USA).

### 2.5. Catalytic Studies

Esterification reactions were performed in a system containing a 250-mL flask equipped with a thermometer, a magnetic stirrer and an inert gas flow. Natural and pillared clays were tested in reactions between 6 and 24 h at 50–120 °C, varying the fatty acid/alcohol molar ratio from 1:1.5–1:2. The relative amount of catalyst in the reaction medium varied between zero and 8 g/100 g of FACO. When using 3A zeolite as the adsorbent, the ratio was varied between 5 and 20 g of adsorbent per 100 g of FACO. Prior to use, the 3A zeolite was activated by placing it in an Edgcon 5P oven with a programmable temperature (300 °C for 24 h) and stored in a desiccator under vacuum until immediately prior to use. This heating procedure was done to allow total desorption of any water molecule present in the zeolite. This procedure does not damage the crystalline structure of the zeolite [30].

In order to separate the zeolites and catalysts from the reaction mixture, the products obtained in the reaction were filtered, and then, the excess alcohol was removed under vacuum in a horizontal distiller (Kugelrohr) at 125 °C. The conversion of FACO to 2-ethylhexyl esters was calculated based on the concentrations of the acids and esters’ fractions obtained by gas chromatography in the product. The samples were analyzed by a Shimadzu Model GC-2010 gas chromatograph, using a DB-5 packed column (20 m × 0.18 mm (internal diameter) × 0.25 μm film). The temperature of the injector was set at 275 °C, and nitrogen was used as the carrier gas. The conversion was calculated considering the identified areas of the fatty acids from castor oil and their esters, respectively.

The reuse of the catalysts was evaluated at mild conditions (50 °C) to clearly detect the difference between each run. Between each cycle, the catalyst/adsorbent were recovered and then washed with hexane and dried at 60 °C.

The products were also analyzed by nuclear magnetic resonance (^1^H-NMR) for certification of the organic ester group formation. The samples were dissolved in deuterated chloroform, and the chemical shifts values were expressed in parts per million (ppm). The 60-MHz Proton NMR spectrometer consists of a Varian EM-360 permanent magnet and a recent upgrade of the electronics and software by Anasazi Instrument (NutsPro version 20021122, New Palestine, IN, USA).

### 2.6. Physicochemical Characterization

Density determinations at 20 °C were performed in a capillary densimeter Model DMA-5000 (Anton Paar, Austria) according to ASTM D4052 [31]. Each sample was run in triplicate, and the average values rounded to the nearest whole degree are reported. The automated Ostwald viscometer (Koehler, New York, NY, USA) was used to measure viscosity according to ASTM D445-17a [31]. Triplicate measurements were carried out, and the average values were reported. The viscosity index (VI), which indicates the effect of temperature on the viscosity, was also calculated. VI values were obtained directly from the kinematic viscosity values at 40 and 100 °C according to ASTM D2270 [31].

The pour point is routinely used to determine the low temperature flow properties of fluids. It evaluates how well the lubricant will perform in low temperature situations. Pour point values were measured according to ASTM D97 [31] using an automatic apparatus CPP 5Gs (ISL, Carpiquet, France). Each sample was run in triplicate, and average values rounded to the nearest whole degree are reported.

Acidity was estimated according to ASTM D664 [31] using the equipment 751 GPD Titrino, Metrohm (Herisau, Switzerland). The automatic titration with potassium hydroxide estimates the total acid number (TAN) as milligrams of KOH per gram of the sample.

## 3. Results and Discussion

### 3.1. Characterization of the Natural and Modified Clays

**Figure 1:**
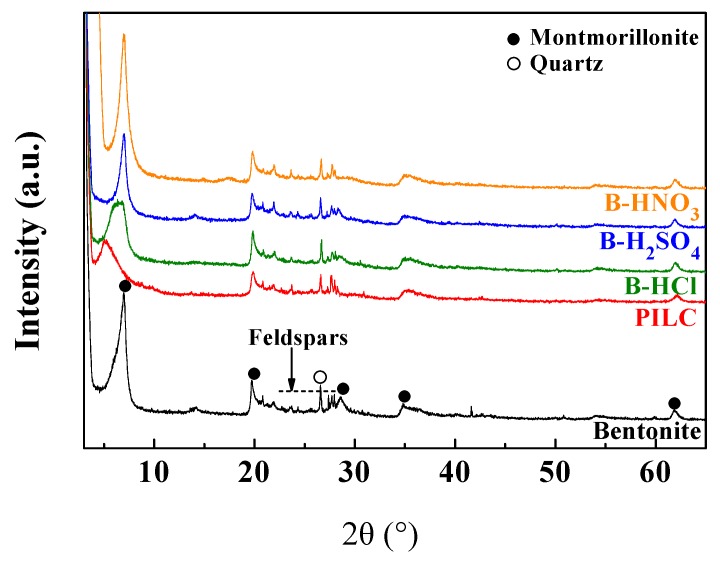
XRD diffractograms of raw bentonite, pillared interlayered clay (PILC), bentonite B-H_2_SO_4_, B-HNO_3_ and B-HCl.

The XRD diffractograms of the raw bentonite and the modified bentonite samples are plotted in Figure 1. The XRD diffractogram of the raw bentonite reveals that a clay mineral, montmorillonite, is the main mineralogical phase. In addition, the presence of minor proportions of quartz and feldspars is noticeable.

The raw bentonite shows a *d*_001_ reflection located at 2θ (°) = 6.97, which leads to a basal spacing of 12.8 Å, suggesting the presence of smectite. This phyllosilicate is composed of two tetrahedral sheet [MO_4_]^4−^ species where M is Si^4+^, Al^3+^ or Fe^3+^. Both tetrahedral sheets, confronted between them, are interconnected through an octahedral sheet where the main cations are Al^3+^, Fe^3+^, Mg^2+^ and Fe^2+^. The tetrahedral-octahedral-tetrahedral (TOT) structure exhibits an excess of the negative charge, which is counterbalanced by the presence of alkaline and alkaline earth cations in the interlayer spacing. The diffractograms of the bentonite treated with H_2_SO_4_ and HNO_3_ show *d*_001_ reflections located at similar 2θ (°) to that shown for the raw bentonite, indicating that the acid treatment does not modify the interlayer spacing. In the case of the bentonite treated with HCl, the *d*_001_ reflection is broader, implying a basal spacing between 12.8 and 14.8 Å. The modification of the interlayer spacing is ascribed to the presence of cations (M^n+^) with different hydration spheres, which implies a modification of the basal spacing.

The *d*_060_ reflection is a determining signal to identify the structure of the smectite. In the case of the raw bentonite, the *d*_060_ reflection peak appears at 2θ (°) = 61.91, which supposes a distance of 1.49 Å. This value is typical of dioctahedral smectites, confirming that the main crystallographic phase is montmorillonite [32]. In this sense, previous authors have pointed out that the acid treatment in dioctahedral smectites is less vulnerable than in the trioctahedral smectites, which are generally rich in Mg^2+^ species [22]. This fact is in agreement with the data shown in Figure 1 where the acid treatment hardly causes damage to the structure of the montmorillonite, since the diffractograms do not show the presence of a broad band between 2θ (°) = 19 and 30 after the acid treatment in any case, which should be ascribed to the formation of amorphous silica [22].

The X-ray diffractogram of the PILC shows how the *d*_001_ reflection is shifted to a lower 2θ value (5.13°), which implies an expansion of the basal spacing to 17.7 Å and confirms the insertion of the Al-polyoxocation in the interlayer spacing [27].

The chemical composition of the catalysts was determined by XRF (Table 1). Considering that the X-ray diffractograms have shown the presence of other crystallographic phases, it is not possible to determine the structural formula of the smectite. From the data shown in Table 1, the presence of M^3+^ species (Al^3+^ and Fe^3+^) may be observed, which confirms the existence of dioctahedral smectite. The higher content of the Al^3+^ species corroborates that the main crystallographic phase must be montmorillonite.

As suggested by the XRD data, the bentonite structure hardly suffers any modifications after the acid treatment. The concentration data of Table 1 show a slight decrease of the Mg^2+^ and Ca^2+^ species by the partial leaching, as well as a decrease of the alkaline species (Na^+^ and K^+^), which are located in the interlayer space, probably due to the exchange of Na^+^ and K^+^ by H^+^ cations. In the case of PILC, the XRF data reveal an increase of the Al_2_O_3_ content. This fact along with the shift of the *d_001_* reflection confirms the insertion of the alumina pillar between the montmorillonite layers.

**Figure 2:**
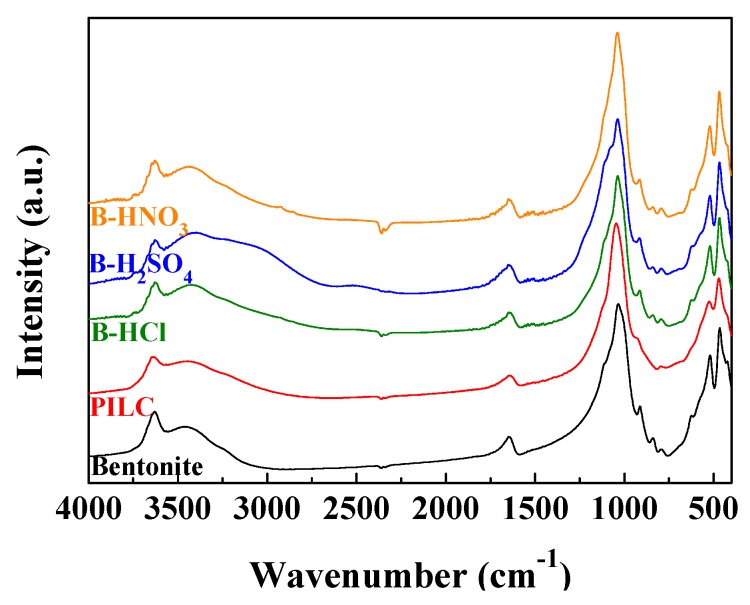
FTIR spectra of raw bentonite, PILC, B-H_2_SO_4_, B-HNO_3_ and B-HCl.

The FTIR spectra of all samples are shown in Figure 2. The –OH stretching bands, located between 3800 and 3500 cm^−1^, are notably influenced by the chemical composition of the octahedral sheet [33]. The spectrum of the raw bentonite sample displays a broad band centered at about 3636 cm^−1^, which is formed by two components located at 3660 cm^−1^ and 3617 cm^−1^ that have been ascribed to Al(OH)Al-stretching vibrations, as previously reported by other authors [22,34]. In addition, the presence of a weak band located at 3732 cm^−1^ related to the OH stretching mode of Si–OH groups is noteworthy. This contribution slightly increases after the acid treatment, which seems to indicate the formation of a small proportion of amorphous silica, although this fact had not been observed from the XRD data [35].

Regarding the bands located between 1800 and 400 cm^−1^ (Figure 2), both the raw bentonite sample and the sample of bentonite treated with acid solutions display similar patterns. This fact suggests that the acid treatment hardly affects the chemical structure of the bentonite, confirming what had been observed in the X-ray diffractograms (Figure 1). All spectra exhibit a main band located at 1035 cm^−1^ attributed to the Si-O stretching of the montmorillonite. The band located about 915 cm^−1^ is ascribed to the presence of Al_2_OH species that are characteristic of dioctahedral smectites [33], while the band located at 790 cm^−1^ is attributed to the Si_2_OH bending mode. The band located at 840 cm^−1^ is assigned to the AlMgOH bending mode. The intensity of this band decreases slightly after the acid treatment. In the same way, the band located at 620 cm^−1^, assigned to the Mg_2_OH bending mode, also decreases with the acid treatment, probably due to a partial leaching of the magnesium species, as previously reported [22]. However, the bands located at 520 and 460 cm^−1^, ascribed to Si–O–Al and Si–O–Si bending vibration modes, hardly suffer modifications after the acid treatment [33]. Finally, the band located at about 1640 cm^−1^ is assigned to H–O–H bending vibration [36]. In the case of the FTIR spectrum of the PILC sample, the intensities of Si–O–Al and Mg–O–Al mode vibrations decrease, even these bands disappear, while the Al–O– bands are maintained.

**Figure 3:**
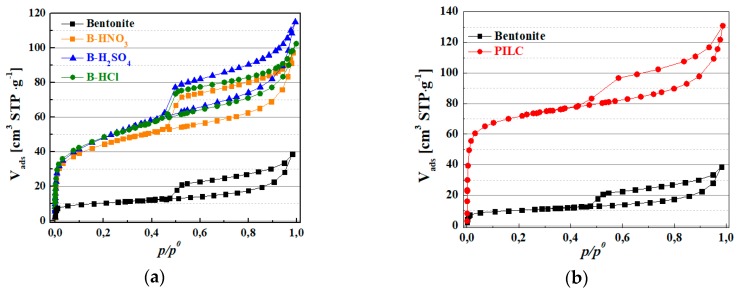
N_2_ adsorption-desorption at −196 °C for raw bentonite: (**a**) B-H_2_SO_4_, B-HNO_3_, B-HCl; (**b**) raw bentonite and PILC.

The N_2_ adsorption-desorption isotherms at −196 °C of raw bentonite, bentonite modified by acid treatments and PILC are compiled in Figure 3. According to the IUPAC classification, these isotherms can be classified as type IIb, in the adsorption branch, with a hysteresis loop indicating the presence of mesopores. In all cases, the presence of a type H4 loop may be observed, which is typical of non-rigid aggregates of plate-like particles (e.g., certain clays) [37]. The bentonite exhibits a small N_2_ adsorption capacity at low relative pressures, related to the low presence of micropores.

The increase in N_2_ adsorption concentration at higher relative pressures, corresponding to the presence of larger mesopores, may be related to voids between particle. All isotherms show an increase of adsorbed N_2_ at lower relative pressures, which indicates an increase of microporosity with the acid treatment. However, the isotherm shape does not exhibit meaningful differences, indicating that the natural clay structure was not significantly modified as revealed from the XRD and FTIR data. This fact suggests the formation of small cavities by the slight leaching of the Mg^2+^ and Ca^2+^ species coming from montmorillonite [22], mainly in the less stacked sheets due to their higher susceptibility to the acid treatment. Regarding the isotherm type of the PILC sample, this may be related to the adsorption in the mono-multilayer region, and it is similar to the raw bentonite sample, suggesting that the interstitial pores and the external structure of the natural clay were not modified by the pillarization process [38]. Furthermore, the formation of the PILC generates micro- and meso-porosity, probably due to a random displacement of the montmorillonite sheets, which generates a house of cards structure, as previously reported by Occelli [39].

The specific surface areas of the catalysts, estimated by the BET equation, are summarized in Table 2. These data reveal how the S_BET_ value increases from 37 m^2^·g^−1^ for the raw bentonite to 158–172 m^2^·g^−1^ for the bentonite modified by acid treatment. Regarding the PILC, the inclusion of alumina pillars between adjacent layers causes a further increase, reaching a S_BET_ value of 270 m^2^·g^−1^.

The total pore volume of the bentonite also increases from 0.04 cm^3^·g^−1^ to 0.12–0.14 cm^3^·g^−1^ for the bentonite treated with acid due to the formation of a higher amount of micropores as previously suggested by the N_2_ adsorption isotherms (Figure 3). For the PILC, the formation of micro- and meso-porosity by the pillars also increases the pore volume from 0.04–0.17 cm^3^·g^−1^.

The higher microporosity of the catalysts could hinder the access of the ricinoleic acid molecule (length ~20 Å) to the active phase, so it is expected that the reaction proceeds on the surface of the non-rigid aggregates of montmorillonite. In the case of the PILC sample, the expansion of the interlayer space is still below the ricinoleic acid length; therefore, it is likely the reaction would also occur on the surface of the PILC. However, the disorder of the structure during the formation of pillars could cause partial delamination and the formation of bigger cavities, which both fatty acids of castor oil (FACO) and 2-ethylhexanol (EH) would access for the esterification reaction to take place.

**Figure 4:**
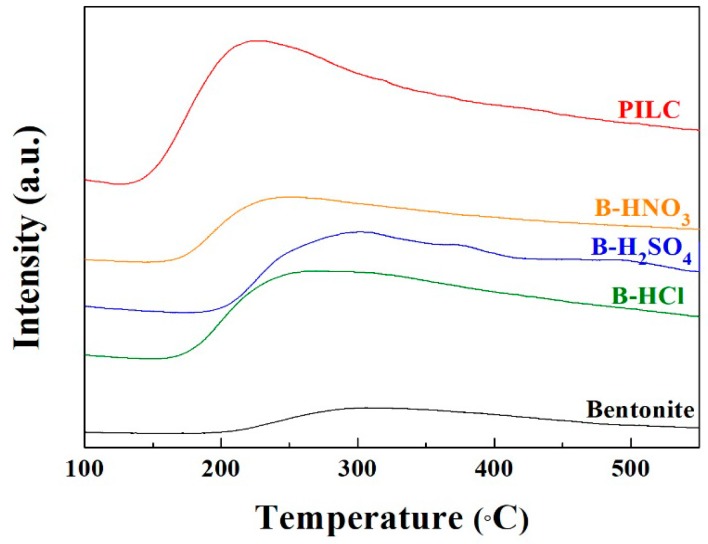
NH_3_-temperature programmed desorption (TPD) of raw bentonite, PILC, B-H_2_SO_4_, B-HNO_3_ and B-HCl.

The ammonia temperature programmed desorption (NH_3_-TPD) allows the determination of the total acid sites of the catalysts (Figure 4). It is well known that the smectite displays an isomorphic substitution Si by Al, generating a charge deficiency, which also generates acid sites that are required by the esterification reaction to obtain biolubricants. The NH_3_ desorption profiles (Figure 4) reveal the presence of acid centers with variable strengths. The raw bentonite exhibits stronger acid sites, although the amount of acid sites is only 86 μmol·g^−1^. From the NH_3_-TPD profiles (Figure 4), the increase of weaker acid sites in the modified materials may be observed in comparison to the raw bentonite. In addition, the formation of microcavities by partial leaching during the acid treatment generates an increase of the charge deficiency, which implies an increase of the available acid sites, obtaining values in the range between 320 and 372 μmol·g^−1^ (see Table 2). In the case of the PILC, the increase of the porosity and the presence of alumina pillars between adjacent layers provide the highest amount of available acid sites (522 μmol·g^−1^) in comparison to the other catalysts, although these sites are also weaker than those shown in the starting bentonite.

### 3.2. Catalytic Results

All samples were evaluated in the esterification reaction of the FACO with EH for the synthesis of potential biolubricants. It is well reported in the literature that the esterification reaction is limited by the molar ratio (alcohol/free fatty acid). The esterification reaction using short-chain alcohols requires an excess of this reagent to shift the reaction towards the products, avoiding the reverse reaction [40]. The esterification reaction with short-chain alcohols follows an Eley–Rideal mechanism since the FACO is only adsorbed on the surface of the catalyst, while the short alcohol directly reacts with it, without adsorption, from the gas phase; therefore the use of high proportions of alcohol to increase the autogenous pressure is necessary [41]. However, the use of large-chain alcohols as the reagent causes a decline in the conversion of the esterification reaction due to several factors such as a decrease in the nucleophilic character of alcohol, steric factors and lower vapor pressure in comparison to shorter alcohols such as methanol [42]. In these cases, it has been reported that higher conversion values are reached with EH:FACO ratios in the range of 1–2 mol/mol [43,44]. In this study, a molar ratio of 1.5 was used in the catalytic tests. Under these conditions, the esterification reaction with large-chain alcohols follows a Hinshelwood–Langmuir mechanism since both alcohol and FACO present similar polarity, favoring the adsorption of both reagents in the same active sites [45].

**Figure 5:**
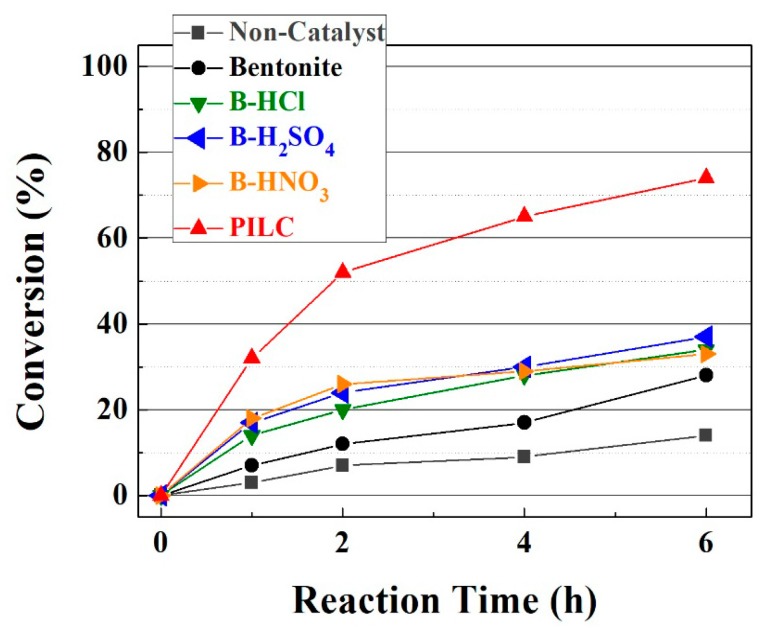
FACO to esters conversion in the esterification reaction of fatty acids from castor oil (FACO) with EH. Catalytic conditions: EH:FACO molar ratio of 1.5, reaction temperature: 50 °C, catalyst loading: 6 g catalyst per 100 g of FACO.

To determine the difference in catalytic behavior between the samples obtained in this study, the experiments were initially performed using a mild temperature (50 °C). It may be seen in Figure 5 that the reaction without catalyst only reaches an FACO conversion to esters of 14 wt % after 6 h.

The incorporation of the raw bentonite as the catalyst in the reaction medium (at a loading of 6 g per 100 g of FACO) only slightly improves the catalytic activity, attaining an FACO to esters conversion of 28 wt % due to the small proportion of acid sites on the surface of the montmorillonite, which favors the esterification reaction. The modification of the raw bentonite with acid treatment hardly increases the FACO to esters conversion (34–38 wt %) after 6 h of reaction. Despite the indication from the NH_3_-TPD analysis of an increase in the available acid sites, these sites are located in micropores where NH_3_ molecules can access, while bulkier molecules such as EH and FACO have their access hindered by steric effects; hence, the esterification reaction to obtain biolubricants must also take place on the surface of the montmorillonite. The catalytic activity of the PILC notably improves those data obtained for raw bentonite and bentonite modified by acid treatment, attaining a FACO to esters conversion of 74 wt %. The higher conversion values are attributed to the expansion of the interlayer spacing (Figure 1), which allows the access of the reagents to the active sites located in the montmorillonite sheets and the Al-pillars [46].

**Figure 6:**
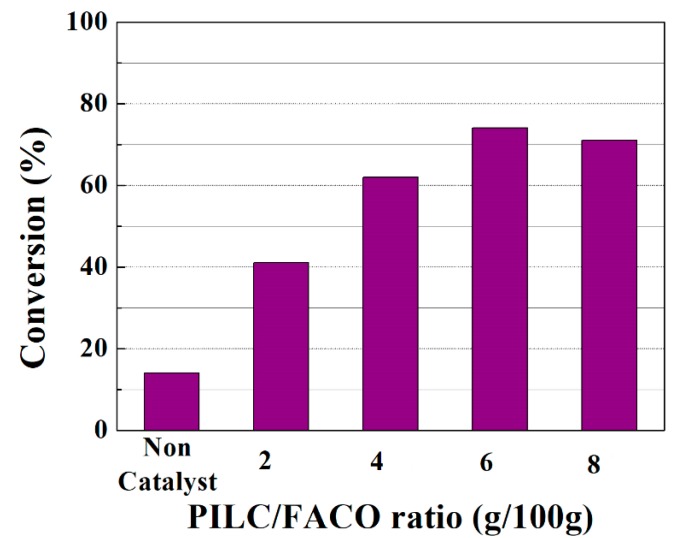
Influence of the PILC/FACO ratio (g/100 g) on the FACO to esters conversion in the esterification reaction of FACO with EH. Catalytic conditions: EH:FACO molar ratio of 1.5, reaction temperature: 50 °C, reaction time: 6 h.

From these results and observing that the PILC sample was the most effective catalyst for the esterification reaction under study, a more detailed study was then performed to evaluate the relative catalytic loading at the same temperature (50 °C). The catalytic results indicate a maximum FACO to esters conversion (ca. 74 wt %) at a catalyst/FACO ratio of 6 g/100 g (see Figure 6) after 6 h of reaction. A higher than 6 PILC to FACO ratio does not improve the FACO conversion values, probably due to mass transfer limitations.

**Figure 7:**
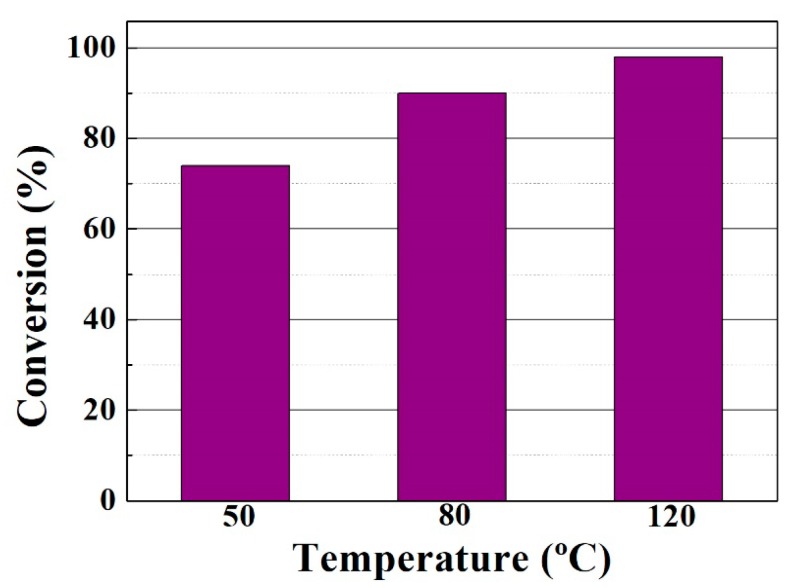
Influence of the reaction temperature on the FACO to esters conversion in the esterification of FACO with EH. Catalytic conditions: EH:FACO molar ratio of 1.5, reaction time: 6 h, catalyst/FACO ratio: 6 g/100 g.

Using this optimized PILCs/FACO ratio of 6 g/100 g, the effect of increasing temperature (up to 120 °C) was evaluated, as seen in Figure 7. The increase in conversion values with increasing temperatures (from 74–98 wt % after 6 h of reaction at 50 and 120 °C, respectively) confirms that the esterification reaction is thermodynamically favored with increasing temperature [47].

**Figure 8:**
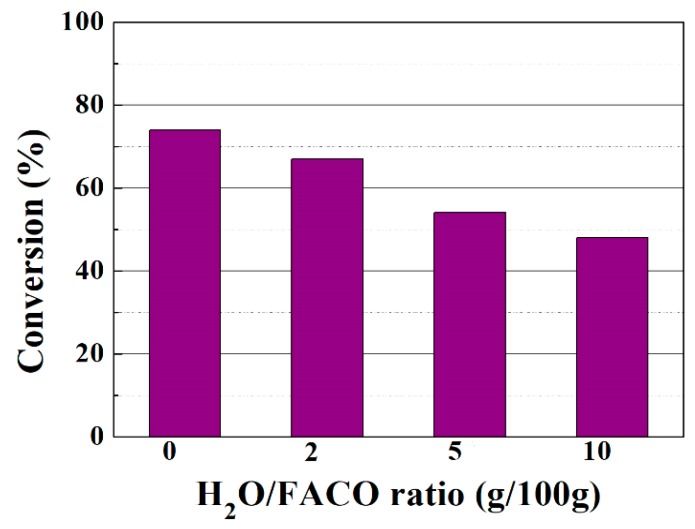
Influence of the H_2_O to FACO ratio (g/100 g) on the FACO to esters conversion in the esterification reaction of FACO with EH. Catalytic conditions: EH:FACO molar ratio of 1.5, reaction temperature: 50 °C, reaction time: 6 h, catalyst to FACO ratio: 6 g/100 g.

Since the esterification reaction also forms H_2_O as a by-product, several authors have pointed out that the presence of H_2_O limits the catalytic conversion due to two factors: on the one hand, H_2_O may adsorb on the active centers of the catalyst, especially at low temperatures; on the other hand, the formation of H_2_O as a product also limits the shift of the thermodynamic equilibrium towards the products [44,48]. Thus, small proportions of H_2_O were added to the reaction medium to evaluate its effect in the FACO reaction to obtain esters (Figure 8). As expected, the incorporation of H_2_O in the reaction medium decreases the FACO to esters conversion from 74 wt % after 6 h of reaction at 50 °C to 48 wt %, when a water to FACO ratio of 10 (g water per 100 g of FACO) is used under similar reaction conditions.

**Figure 9:**
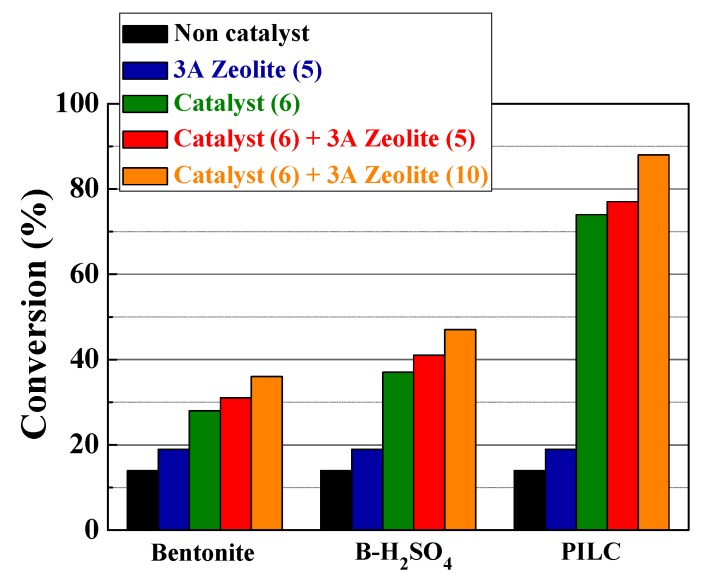
Influence of increasing ratio of 3A zeolite (g/100 g of FACO) on the FACO to esters conversion in the esterification reaction of FACO with EH using PILC. Catalytic conditions: EH:FACO molar ratio of 1.5, reaction temperature: 50 °C, reaction time: 6 h, catalyst to FACO ratio: 6 g/100 g.

In an attempt to minimize the decrease of the catalytic activity by the presence of H_2_O as a by-product, 3A zeolite was used as the adsorbent to retain the H_2_O molecules formed in the esterification reaction. In a preliminary test, the FACO esterification was carried out only with the addition of 5 g of 3A zeolite per 100 g of FACO, without clay mineral or PILC (see Figure 9). It may be observed that only with the use of 3A zeolite, there is an improvement of the FACO to esters conversions obtained for the reaction without catalyst (roughly a 3–5% increase in conversion values). Despite the fact that the zeolite possesses potentially active acidic sites for esterification reactions, the small pore size of the 3A zeolite (3Å) prevents the access of the FACO and EH to the micropores, so the slight increase of the catalytic activity is assigned to the small proportion of acid sites located on the surface of the 3A zeolite. Based on these results, both the catalyst and 3A zeolite as the adsorbent were simultaneously added to the reaction medium. The FACO to esters conversion values, also shown in Figure 9, reveal the resulting improvement as 3A zeolite content increases, attaining a maximum FACO esterification of 88 wt % after 6 h of reaction at 50 °C when 10 g of 3A zeolite per 100 g of FACO were added to the reaction medium. This suggests that the 3A zeolite indeed retains H_2_O molecules obtained as a by-product of the esterification reaction, shifting the reaction towards the products reaching higher FACO to esters conversion values. A similar trend was also observed for the raw bentonite, as well as for the bentonite after the acid treatment, although the conversion of FACO was still limited by the textural properties of the clay, as seen before.

**Figure 10:**
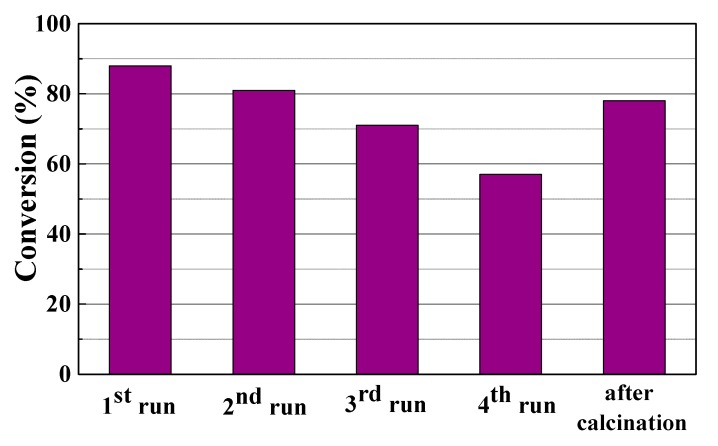
Reuse of the PILC as catalyst in the esterification reaction between FACO and EH after four reaction cycles and after calcination at 500 °C. Catalytic conditions: EH:FACO molar ratio of 1.5, reaction temperature: 50 °C, reaction time: 6 h, catalyst to FACO ratio: 6 g/100 g, zeolite 3A to FACO ratio: 10 g/100 g.

Although these catalysts are relatively inexpensive, the reuse of catalysts is a key factor to achieve a sustainable process. The catalytic data, shown in Figure 10, reveal that the FACO to esters conversion decreases with the number of cycles. This decrease in catalytic activity may be ascribed on the one hand to the formation of carbonaceous deposits that block active acid sites involved in the esterification reaction and on the other hand to the H_2_O molecules, obtained as a by-product in the esterification reaction, which saturate the nanocavities of the 3A zeolite, disabling its capacity as the adsorbent. Taking into account the progressive deactivation after several reaction cycles, both catalyst and adsorbent were regenerated by calcination at 500 °C for 4 h. The obtained data, also compiled in Figure 10, show that the conversion value is slightly lower than that obtained for the fresh catalyst. This may be attributed to some loss of catalyst and adsorbent in the treatments between each cycle.

Another key parameter to obtain sustainable heterogeneous catalysts in batch experiments is the evaluation of leaching in the reaction medium. Although the elements that could be leached (Si, Al or Mg) are innocuous for the environment and humans, the liquid obtained after the reaction was collected and analyzed by ICP-MS. The leaching of PILC may be considered as negligible since the leaching in the reaction medium after the first cycle was only 0.005 wt % for Si^4+^, 0.006 wt % for Al^3+^ and 0.010 wt % for Mg^2+^. The low leaching may be attributed to the low polarity of both FACO and EH in comparison to the synthesis of biodiesel, where a larger proportion of short alkyl-chain alcohol with a higher polarity is required [49].

### 3.3. Physicochemical Properties and Chemical Characterization

Considering that the aim of the present research was the synthesis of potential biolubricants from the esterification reaction between the EH and FACO, the physicochemical properties of the 2-ethylhexyl esters were evaluated.

Pour point is an important parameter to define the potential of a biolubricant. Using fatty acids has great potential for biolubricant synthesis since vegetable oils usually display lower pour points than traditional mineral oils. From the pour point data, shown in Table 3, it is noteworthy that the synthesis of the branched esters slightly decreases the pour temperature. In any case, the low pour point favors their use as biolubricants, maintaining their interesting properties even in winter conditions.

The viscosity index is another key parameter to obtain an appropriate lubricant. Both castor oil and FACO tend to interact with other molecules by hydrogen bonds [50,51], making useless their use for industrial applications. The esterification reaction of the FACO with a branched alcohol causes a drastic decrease in viscosity at low temperature (40 °C) compared to castor oil or FACO, which improves its fluidity to be used as a biolubricant since the ester would be easier to pump and achieve finer droplets [50].

The high densities of the castor oil, FACO and its obtained esters, as seen in Table 3, may be attributed to the presence of the OH– group in the free fatty acid chain. Regarding the total acid number (TAN), the obtained ester displays a lower TAN value than castor oil and FACO, as expected.

The flash point is the temperature at which a compound can ignite when it is exposed; therefore, the flash point may be considered as a key parameter from the point of view of safe handling, storage and transport. The obtained esters display a flash point temperature of 205 °C. This high value is attributed to the long chain of the FACO and the alcohol, as well as the presence of the –OH group in the ricinoleic acid, which favors the formation of hydrogen bonds. Taking into account these values, the obtained esters product is a non-hazardous additive, which may be used to increase the flash point of a mineral lubricant.

**Figure 11:**
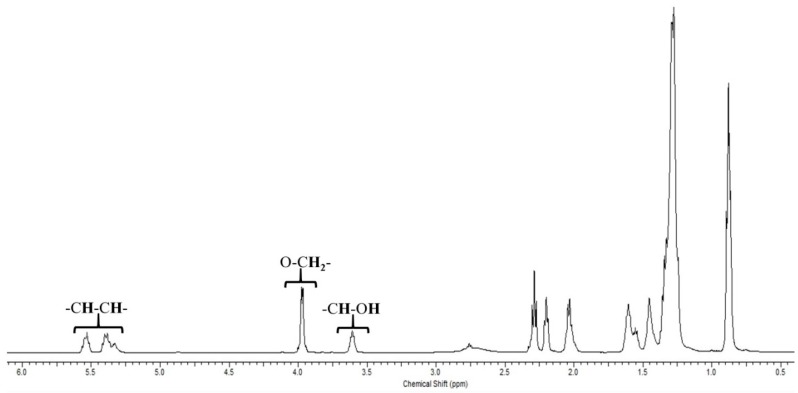
^1^H NMR spectrum of the obtained ester after the esterification reaction between FACO and EH.

Finally, the structure of the obtained esters was determined from its ^1^H NMR spectrum (Figure 11). The chemical shift located at about δ: 3.61 ppm displays the existence of the –CH–OH, which discards the reaction of the FACO with the –OH group of another ricinoleic acid molecule. In the same way, the chemical shift centered at about δ: 3.95 ppm is assigned to –O–CH_2_– species, confirming the esterification reaction between the FACO and EH.

## 4. Conclusions

Natural and modified bentonite clays were evaluated as heterogeneous catalysts in the esterification reaction of the fatty acids from castor oil with 2-ethylhexanol for bio-based lubricant synthesis. Raw bentonite only slightly improved the FACO to esters conversion of 14 wt % for the reaction without catalyst to 28 wt % with raw bentonite, after 6 h under mild reaction conditions. Modifications of the raw bentonite with acid treatments slightly increased the FACO to esters conversion (34–38%) after 6 h of reaction. However, when using pillared clays, the FACO to esters conversion notably increased to values around 74 wt %. These higher conversion values are attributed to the expansion of the interlayer spacing, which allows the access of the reagents to the active sites located in the montmorillonite sheets and the Al-pillars. To evaluate the influence of H_2_O on catalytic activity at lower temperatures, both catalyst and 3A zeolite as adsorbent were simultaneously added to the reaction medium. The FACO to esters conversion improved as the 3A zeolite concentration increased at the reaction medium, attaining a maximum FACO esterification of 88 wt % after 6 h of reaction at 50 °C using a ratio of 10 g of 3A zeolite to 100 g of FACO. These results suggest that the adsorbent seems to retain the H_2_O molecules, obtained as a by-product of the esterification reaction, shifting the reaction equilibrium towards the products reaching higher conversion values.

## Figures and Tables

**Table 1:** Chemical analysis, estimated by XRF, of the raw bentonite, bentonite modified by acid treatment and PILC (wt %).

Sample	Concentration (wt %)									
	SiO_2_	Al_2_O_3_	Fe_2_O_3_	MgO	Na_2_O	K_2_O	CaO	MnO	P_2_O_5_	TiO_2_
Bentonite	66.85	20.51	4.06	4.11	1.65	0.60	1.67	0.03	0.08	0.15
B-HNO_3_	68.13	22.29	4.03	3.91	0.49	0.42	0.35	0.02	0.02	0.15
B-H_2_SO_4_	68.40	22.23	3.87	3.95	0.35	0.37	0.24	0.02	0.05	0.15
B-HCl	68.42	22.40	4.02	3.85	0.39	0.41	0.28	0.01	0.04	0.15
PILC	59.80	33.18	3.32	3.30	0.25	0.29	0.22	0.01	0.02	0.12

**Table 2:** Properties of raw bentonite, bentonite modified by acid treatment and PILC.

Samples	S_BET_ (m^2^·g^−1^)	V_µP_ (cm^3^·g^−1^)	V_TP_ (cm^3^·g^−1^)	Amount Acid Sites ^1^ (μmol·g^−1^)
Bentonite	37	0.01	0.04	145
B-H_2_SO_4_	171	0.05	0.14	357
B-HNO_3_	158	0.05	0.12	320
B-HCl	172	0.05	0.13	374
PILC	270	0.10	0.17	522

^1^ Determination of acid sites from NH_3_-TPD experiments.

**Table 3:** Physicochemical properties of castor oil, FACO and obtained ester.

Samples	Density at 20 °C (g.cm^−3^)	Pour Point (°C)	Flash Point (°C)	TAN	Viscosity (cSt)		Viscosity Index
				(mg KOH.g^−1^)	40 °C	100 °C	
Castor Oil	0.9582	−15	145	1.19	261.3	19.60	84
FACO	0.9393	−36	180	127	132.2	13.0	90
Esters	0.9084	−39	205	0.61	38.4	7.29	157
